# Prevalence of cognitive impairment in patients with rheumatoid arthritis: a cross sectional study

**DOI:** 10.1186/s12888-022-04417-w

**Published:** 2022-12-09

**Authors:** Bethany McDowell, Calum Marr, Clive Holmes, Christopher J. Edwards, Christopher Cardwell, Michelle McHenry, Gary Meenagh, Bernadette McGuinness

**Affiliations:** 1 Queen’s University Belfast, Belfast, Northern Ireland; 2 University of Southampton, Southampton, England; 3 Southern Health NHS Foundation Trust, Southampton, England; 4 NIHR Southampton Clinical Research Facility, Southampton, England; 5 University Hospital Southampton NHS Foundation Trust, Southampton, England; 6 Belfast Health and Social Care Trust, Belfast, Northern Ireland; 7 Northern Health and Social Care Trust, Antrim, Northern Ireland

**Keywords:** Rheumatoid arthritis, Mild cognitive impairment, Inflammation

## Abstract

**Objective:**

To explore the role of chronic inflammation in rheumatoid arthritis (RA) on cognition.

**Methods and analysis:**

Six hundred sixty-one men and women aged ≥55 years who fulfilled the American College of Rheumatology/European League Against Rheumatism (ACR/EULAR) criteria for RA were recruited from three healthcare trusts in the United Kingdom (UK) between May 2018 and March 2020. Study participants took part in interviews which captured sociodemographic information, followed by an assessment of cognition. RA specific clinical characteristics were obtained from hospital medical records. Participants were cognitively assessed using the Montreal Cognitive Assessment (MoCA) and were classified as cognitively impaired if they scored ≤27/30 points. Linear regression analyses were conducted to identify which demographic and clinical variables were potential predictors of cognitive impairment.

**Results:**

The average age of participants was 67.6 years and 67% (444/661) were women. 72% (458/634; 95% CI 0.69 to 0.76) of participants were classified as cognitively impaired (MoCA≤27). Greater cognitive impairment was associated with older age (*p* = .006), being male (*p* = .041) and higher disease activity score (DAS28) (with moderate (DAS28 > 3.1) (*p* = 0.008) and high (DAS28 > 5.1) (*p* = 0.008)) compared to those in remission (DAS28 ≤ 2.6). There was no association between MoCA score and education, disease duration, RF status, anti-cyclic citrullinated peptide (anti-CCP) status, RA medication type or use of glucocorticoids or non-steroidal anti-inflammatory drugs (*p* > 0.05).

**Conclusion:**

This study suggests that cognitive impairment is highly prevalent in older adults with RA. This impairment appears to be associated with higher RA disease activity and supports the concept that chronic systemic inflammation might accelerate cognitive decline. This underlines the importance of controlling the inflammatory response.

**Supplementary Information:**

The online version contains supplementary material available at 10.1186/s12888-022-04417-w.

## Introduction

Rheumatoid arthritis (RA) is a chronic inflammatory autoimmune disease affecting approximately 0.5–1% of the global population but is more common in women [1, 2]. The main characteristic of RA is persistent inflammation in the joint synovium [3]. Without early and effective management, RA can cause progressive joint damage resulting in reduced functional capacity and quality of life [4–6]. Disease Modifying Anti-Rheumatic Drugs (DMARDs) are used to reduce disease activity and joint destruction. Different types of DMARDs exist including conventional synthetic (cs) DMARDs, biologic (b) DMARDs and targeted synthetic (ts) DMARDs [7, 8]. RA is also associated with damage as a result of chronic systemic inflammation. This includes accelerated cardiovascular disease and stroke [9–11]. As a result, RA appears to be an ideal disease to study biologically to understand the accelerating effects of inflammation in a number of disease areas, including cognitive decline associated with dementia [12].

In recent years there has been increasing interest in the potential role of systemic inflammation in the pathogenesis of Alzheimer’s disease (AD) [13, 14]. This is of particular relevance in RA as these patients have higher levels of systemic inflammation than the general population, therefore, if AD is driven by inflammatory processes then these patients may be at an increased risk of cognitive impairment and dementia as has been suggested by recent epidemiological studies [15–18]. There are several small studies that have highlighted the burden of cognitive impairment in RA [19–22]. A systematic review sought to explore the prevalence of cognitive impairment in RA and found that individuals with RA significantly underperform in cognitive assessments compared to healthy controls and also identified age, education, disease activity and depression as factors associated with cognitive impairment, although this was inconsistent across individual studies and based on small sample sizes ranging from 13 to 157 participants [23].

As intact cognitive function in RA is also essential for successfully performing day-to-day activities and adhering to treatment programs, understanding the biological and clinical factors which may affect cognition in this condition is important for improving clinical outcomes and quality of life [24]. The purpose of this study was to investigate the prevalence of cognitive impairment in a larger population of older UK individuals with RA and identify specific factors that may be associated with cognitive impairment.

## Methods

### Sample and setting

Participants were recruited on behalf of the rheumatoid arthritis medication and memory study (RESIST), a multicentre longitudinal observational study which aims to compare cognitive decline in adults with both RA and mild cognitive impairment (MCI) who are on conventional synthetic disease modifying antirheumatic drugs (csDMARDs) to adults with both RA and MCI who are on a Tumour Necrosis Factor Inhibitor (TNFi). The primary objective is to determine if TNFi treatment reduces the rate of cognitive decline compared to csDMARDs in patients with RA and MCI. Results from the longitudinal study will be important in further describing the role of inflammation in the pathogenesis of AD and will help to determine the potential of TNFi in prevention of cognitive decline. All data presented here was collected during baseline screening interviews, prior to enrolment on the longitudinal phase of the study. We used the Strengthening the Reporting of Observational Studies in Epidemiology (STROBE) cross sectional checklist when writing our report [25].

Participants were recruited between May 2018 and March 2020 during their regularly scheduled check-up appointments in rheumatology outpatient clinics within the Belfast Health and Social Care Trust (BHSCT) located in the Royal Victoria Hospital, Belfast City Hospital, and Musgrave Park Hospital; Northern Health and Social Care Trust (NHSCT) located in Antrim Area Hospital; and University Hospital Southampton NHS Foundation Trust (UHS) located in Southampton General Hospital.

All individuals older than 54 years of age who fulfilled the American College of Rheumatology/European League Against Rheumatism (ACR/EULAR) criteria for RA [26] (regardless of disease duration) were identified and approached for inclusion in the study by rheumatology consultants and specialist rheumatology nurses.

### Study procedures

Participants took part in face-to-face interviews with trained research members after providing written informed consent. Interviews captured sociodemographic information including age, sex and education. Disease related characteristics, including a disease activity score for RA (DAS28) upon commencement of treatment, were obtained from medical records where available. Participants indicated the date (or approximate date) of their RA diagnosis, which was used to calculate a measure of disease duration (in years). This was confirmed in clinical records, if available.

DAS28 calculates a score based on the number of tender and swollen joints, a visual analogue scale global assessment of health, and acute phase reactants – either erythrocyte sedimentation rate (ESR) or C-reactive protein (CRP) titre level [27]. In the present study DAS28-ESR was used. Disease activity was categorised based on the following scores: remission (< 2.6), low activity (2.6–3.2), moderate activity (3.2–5.1), or high activity (> 5.1) [28].

Anti-cyclic citrullinated peptide (anti-CCP) and rheumatoid factor (RF) are autoantibodies associated with RA and were also investigated as a measure of seropositivity.

Participants were classified as taking either a csDMARD, a TNFi, or a combination of both. Participants also provided a list of any other medication they were currently taking. From this information, two additional variables were computed: glucocorticoid use (specifically use of prednisolone; participants were classified as either taking or not taking) and non-steroidal anti-inflammatory drug (NSAID) use (participants were classified as taking or not taking any of the following; aspirin, diclofenac, ibuprofen or naproxen).

Participants were cognitively assessed using the Montreal Cognitive Assessment (MoCA) (Version 7.1). The MoCA was developed as a screening tool for the detection of MCI and has been widely validated for use in both clinical and research settings [29–33]. It is a 10-min, 30-point pen and paper test which assesses multiple cognitive domains including visuospatial skills, executive function, language, memory (delayed recall), attention, concentration, abstraction, and orientation. To account for differences in educational attainment an additional one point was awarded if the subject had spent ≤12 years in full-time education. Cognitive impairment was defined by a cut-off score of ≤27/30 pts. which has previously demonstrated good sensitivity for detection of MCI [34, 35].

### Statistics

Participant characteristics were summarised using means and standard deviations (SD) for continuous variables and relative frequencies and percentages for categorical variables. The proportion of participants with cognitive impairment was determined and a linear regression model was created to identify variables associated with MoCA score. The model included variables which have been previously linked with cognitive impairment in the context of RA including age, sex, education (≤12 years or > 12 years), disease duration, disease activity (remission, low, moderate, or high), RF status (positive or negative), anti-CCP status (positive or negative), RA medication type, and use of glucocorticoids and NSAIDs.

Linear regression was used to avoid information and power loss associated with dichotomising the continuous variable of MoCA score into the categories of cognitively impaired/non-impaired. However, for consistency, we also conducted logistic regression using the same predictors to examine whether they were associated with the likelihood of a participant being classified as impaired (MoCA ≤27/30).

It is likely that other confounding variables that were not collected at the screening stage would also have an influence on cognitive function (e.g. pain, depression, physical impairment). Data on these variables was collected later during the longitudinal phase of the study and hence could not be included in baseline analyses. All analyses were conducted using SPSS, version 27.0.

### Patient and public involvement

Members of the public were involved in the design and preparation of the RESIST study proposal, specifically in wording and logistics. We also received advice when designing study documents including the participant information sheet, consent forms, and protocol. Research Network volunteers from the Alzheimer’s Society assigned to the project were invited into the project advisory committee and advised on all aspects of the project.

## Results

A total of 720 participants were interviewed between the 22nd May 2018 and the 10th March 2020. *N* = 59 participants were excluded as they did not meet inclusion criteria (*n* = 54 participants were not on a csDMARD or TNFi, *n* = 3 participants did not have a diagnosis of RA, *n* = 1 participant was under 55 years old, and *n* = 1 participant had an incomplete MoCA). Therefore 661 participants were included in this analysis. Participant sociodemographic and clinical characteristics are presented in Table 1. The mean age of participants was 67.6 years (SD 8.1), 67% were women and 37% had > 12 schooling years. RA clinical characteristics were obtained from medical records and thus were not always available. Data on disease duration was available for *n* = 556 participants, DAS28 score was available for *n* = 337 participants, RF status was available for *n* = 437 and anti-CCP status was available for *n* = 423. Five hundred ninety-two participants provided information on any medication they were taking at the time of screening.

**Table 1:** Summary of participant demographic and clinical characteristics (*n* = 661)^a^

	Total	Cognitively Impaired (MoCA ≤27/30)	Non-impaired (MoCA > 27/30)	*P* value†
N	661	458	176	
Age (years)	67.6 (8.1) [55, 89]	68.8 (8.4) [55, 89]	64.7 (6.5) [55, 85]	**<.001**
Sex				.158
*Male*	217 (32.8)	157 (34.3)	50 (28.4)	
*Female*	444 (67.2)	301 (65.7)	126 (71.6)	
Educational attainment				**<.001**
*≤ 12 years*	386 (58.4)	306 (66.8)	80 (46.2)	
*> 12 years*	245 (37.1)	152 (33.2)	93 (53.8)	
*Missing*	30 (4.5)	–	–	
ESR (mm/hr) (*n* = 532)	22.6 (21.1) [1, 137]	23.7 (21.4) [1, 137]	17.1 (16.9) [1, 105]	**.002**
DAS28-ESRscore (*n* = 337)	3.5 (1.5) [0.63, 7.85]	3.6 (1.5) [0.63, 7.85]	3.1 (1.3) [1.21, 6.74]	**.007**
Disease duration (years) (*n* = 556)	12.6 (12.9) [0, 73]	12.8 (13.2) [0, 73]	12.2 (12.0) [0, 60]	.618
Disease activity				.079
*Remission (DAS28 ≤ 2.6)*	92 (13.9)	61 (13.3)	31 (17.6)	
*Low (2.6 < DAS ≤ 3.2)*	60 (9.1)	38 (8.3)	21 (11.9)	
*Moderate (3.2 < DAS ≤ 5.1)*	135 (20.4)	101 (21.1)	31 (17.6)	
*High (DAS > 5.1)*	50 (7.6)	40 (8.7)	9 (5.1)	
*Missing*	324 (49.0)	218 (47.6)	84 (47.7)	
Anti-CCP				.505
*Positive*	267 (40.4)	201 (43.9)	56 (31.8)	
*Negative*	156 (23.6)	113 (24.7)	37 (21.0)	
*Missing*	238 (36.0)	144 (31.4)	83 (47.2)	
RF				.141
*Positive*	300 (45.4)	223 (48.7)	68 (38.6)	
*Negative*	137 (20.7)	93 (20.3)	40 (22.7)	
*Missing*	224 (33.9)	142 (31.0)	68 (38.6)	
RA Medication				.731
*csDMARD + TNFi*	125 (18.9)	97 (21.2)	26 (14.8)	
*csDMARD only*	393 (59.5)	293 (64.0)	86 (48.9)	
*TNFi only*	74 (11.2)	54 (11.8)	19 (10.8)	
*Missing*	69 (10.4)	14 (3.1)	45 (25.6)	
Taking prednisolone				.743
*Yes*	37 (5.6)	27 (5.9)	9 (5.1)	
*No*	555 (84.0)	417 (91.0)	122 (69.3)	
*Missing*	69 (10.4)	14 (3.1)	45 (25.6)	
Taking NSAID				.149
*Yes*	80 (12.1)	56 (12.2)	23 (13.1)	
*No*	512 (77.5)	388 (84.7)	108 (61.4)	
*Missing*	69 (10.4)	14 (3.1)	45 (25.6)	

^a^Demographics are summarised as mean (SD) [min, max] for continuous variables or frequency (%) for categorical variables

^†^Independent samples T-tests were used to compare continuous variables and Chi-squared tests were used for categorical variables. *P* values were significant at *p* < 0.05

Education-adjusted MoCA score was available for *n* = 634 participants at the time of analysis. The mean education-adjusted MoCA score was 25.4 (SD 3.1). The proportion of participants who were classified as cognitively impaired according to a cut off of ≤27/30 was 72.2% (458/634; 95% CI 0.69 to 0.76). Those who were cognitively impaired, on average were older (68.8 years versus 64.7 years), had higher ESR measurements (23.7 mm/hr. versus 17.1 mm/hr), higher DAS28 scores (3.6 versus 3.1) and were more likely to have spent 12 years or less in full-time education (66.8% versus 46.2%). Disease duration did not significantly differ between those who were classified as cognitively impaired and those who were not.

A MoCA cut-off score of < 26/30 is also widely used for detection of MCI, demonstrating good sensitivity and specificity [32–36]. For comparison, when we used this cut-off score, 45.7% (290/634; 95% CI 0.42 to 0.50) of our population was classified as cognitively impaired. However, we elected to use a cut-off of ≤27/30 in all of our analyses as participants in the present sample were aged just 55 years and older (mean 67.6 years) and were not recruited from memory clinics.

The associations between MoCA score and various characteristics are presented in Table 2. A multivariable regression model with age, sex, education, RA disease activity, RF status, anti-CCP status, RA medication type, glucocorticoid (prednisolone) use and NSAID use as independent variables explained 23% of the variance in MoCA score (*F*(13, 202) = 4.57, *p* < .001, R^2^ = 0.23, adjusted R^2^ = 0.18). Linear regression analysis revealed that increasing age (*p* = .006) was associated with lower MoCA score. After controlling for the other variables in the model, per 10-year increase in age, MoCA was on average 0.58 (95% CI 0.17, 1.00) points lower. Sex was also associated with MoCA score, with females scoring 0.74 (95% CI 0.03, 1.45) points higher than males.

**Table 2:** Summary of linear regression analyses with MoCA score as the outcome

Independent variable	Simple Linear Regression		Multiple Linear Regression	
	β Coefficient (95% CI)	*P* value	β Coefficient (95% CI)	Adjusted *P* value
Age (per 10-year increase)	−1.13 (− 1.42, − 0.84)	<.001^*^	− 0.58 (− 1.00, − 0.17)	.006^*^
Sex				
*Male*	0.00 (reference)	–	0.00 (reference)	–
*Female*	0.52 (−0.01, 1.04)	.053	0.74 (0.03, 1.45)	.041^*^
Education				
*≤ 12 years*	0.00 (reference)	–	0.00 (reference)	–
*> 12 years*	1.46 (0.97, 1.95)	<.001^*^	0.51 (−0.18, 1.20)	.149
Disease duration	0.01 (−0.01, 0.03)	.496	0.03 (−0.01, 0.06)	.119
Disease severity				
*Remission (DAS28 ≤ 2.6)*	0.00 (reference)	–	0.00 (reference)	–
*Low (2.61 < DAS28 ≤ 3.2)*	−0.33 (−1.27, 0.62)	.497	0.60 (− 0.48, 1.68)	.275
*Moderate (3.21 < DAS28 ≤ 5.1)*	−0.95 (− 1.72, − 0.18)	.016^*^	− 1.10 (− 1.90, − 0.30)	.008^*^
*High (DAS28 > 5.1)*	−1.33 (− 2.34, − 0.33)	.009^*^	−1.41 (− 2.45, − 0.36)	.008^*^
RF				
*Negative*	0.00 (reference)	–	0.00 (reference)	–
*Positive*	−0.68 (−1.28, − 0.09)	.025^*^	−0.68 (− 1.61, 0.25)	.150
Anti-CCP				
*Negative*	0.00 (reference)	–	0.00 (reference)	–
*Positive*	−0.36 (− 0.94, 0.22)	.225	− 0.76 (− 1.66, 0.14)	.097
RA Medication				
*csDMARD + TNFi*	0.00 (reference)	–	0.00 (reference)	–
*csDMARD only*	0.41 (−0.19, 1.02)	.183	0.25 (−0.68, 1.17)	.601
*TNFi only*	0.55 (− 0.31, 1.41)	.208	0.72 (−0.91, 2.34)	.387
Taking prednisolone				
*No*	0.00 (reference)	–	0.00 (reference)	–
*Yes*	−0.14 (−1.14, 0.87)	.788	0.86 (−0.70, 2.42)	.277
Taking NSAID				
*No*	0.00 (reference)	–	0.00 (reference)	–
*Yes*	− 0.15 (− 0.86, 0.56)	.679	0.97 (− 0.01, 1.95)	.053

^*^Significant at the 5% level, *p* < 0.05

On linear regression, participants with moderate (*p =* .008) and high (*p* = .008) levels of disease activity had lower MoCA scores by on average 1.10 units (95% CI 0.30, 1.90) and 1.41 units (95% CI 0.36, 2.45), respectively, compared with those in remission after adjustments. Low disease activity did not appear to affect cognition compared to those in remission (*p* = .275). Neither RF nor anti-CCP status was associated with cognition after adjustment for other variables in the model (*ps* > .05). RA medication was not associated with MoCA score; those taking both a csDMARD and a TNFi did not show significant differences in score compared to those taking csDMARDs only (*p* = .601) or those taking TNFis only (*p* = .387). Neither prednisolone use nor NSAID use was associated with MoCA score (*ps* > .05). Age, disease severity and NSAID use were significantly associated with the likelihood of participants being classified as cognitively impaired (MoCA ≤27/30) in a logistic regression model (see Additional file 1).

## Discussion

This study produced descriptive data for a large sample of older adults with RA that were cognitively assessed using the MoCA and investigated the prevalence and potential predictors of cognitive impairment in this population. Over two thirds (72.2%) of our participants were classified as cognitively impaired using a MoCA cut-off score of ≤27/30 points.

Our findings are analogous to previous studies which assessed cognitive function in patients with RA. A study by Bartolini et al. [20] reported cognitive impairment in 38–71% of their RA cohort with worst cognitive outcomes observed in the visuospatial/executive function tasks. A study by Shin et al. [21] observed that cognitive impairment in RA ranged from 8% on semantic fluency tasks to 29% on visuospatial/memory tasks with 31% of their cohort being classified as cognitively impaired overall. Similarly, Appenzeller et al. [19] found 30% of their RA cohort to be cognitively impaired in a battery of neuropsychological assessments. We found lower prevalence rates compared to a recent study by Vitturi et al. [22] who observed cognitive impairment in 98% of their RA population using a cut-off score of 26/30 in the MoCA. This discrepancy may be due to low educational attainment in that study as 46% of RA participants had less than 4 schooling years and 3% were illiterate. The main limitation of these studies is that they consisted of small RA sample sizes [19, 20, 22]. While methodology varies between these studies making direct comparisons difficult, they imply that the burden of cognitive impairment in RA is significant and highlights the need for standardised longitudinal assessment of cognition in these patients.

In this study, age was identified as a non-modifiable demographic factor that was associated with cognitive impairment. Age is known to be the biggest risk factor for AD and has been negatively correlated with MoCA performance in many previous studies, thus this result was anticipated [36–41].

We demonstrated an inverse linear relationship between DAS28 score and MoCA score which is analogous to previous studies [42, 43]. Moreover, we found that moderate and high disease activity were associated with worse cognitive outcomes compared to those in remission. Together, these findings support the hypothesis that higher levels of chronic systemic inflammation significantly affect cognitive function [44, 45] which has implications beyond the scope of RA. Thus strategies to prevent cognitive impairment and reduce risk of AD should include measures to modulate inflammation.

It has been hypothesised that anti-inflammatory drugs often prescribed for RA may be beneficial in slowing or preventing cognitive decline [12]. Those taking NSAIDs in the present study were less likely to be classified as cognitively impaired in a logistic regression model. Although results from initial observational studies suggested that NSAID use was associated with a reduced risk of dementia [46–48], there has been little evidence of cognitive benefit in subsequent clinical trials [49–53]. There are now several studies which have suggested the potential benefits of DMARDs on cognition [16, 18, 54–57], but this requires further investigation in longitudinal studies.

This study identified several limitations that should be taken into consideration when interpreting the results. This study was cross-sectional and as such cannot imply a cause-effect relationship between variables and cognitive outcome. Additionally, no control group was included in the study design, therefore prevalence of cognitive impairment in RA could not be compared to a healthy population or other clinical group. As noted in the introduction, however, a recent systematic review found that individuals with RA significantly underperform in cognitive assessments compared to healthy controls [23]. Cognitive function was assessed using the MoCA alone and the sensitivity and specificity of the cut-off score used was not able to be evaluated due to the lack of known MCI diagnoses. Therefore, the possibility of false positives cannot be dismissed and will be further investigated in the longitudinal RESIST study. We did not record domain level scores from the MoCA. It is possible that inflammation may have stronger associations with performance in specific cognitive domains such as memory or processing speed; future studies should investigate this possibility further by including domain specific measures of cognitive function. As part of the eligibility criteria for the RESIST study, only participants who were on a csDMARD or TNFi at the time of enrolment were considered in this analysis which may limit the generalisability of our findings. Additionally, there was a large amount of missing data in relation to RA disease characteristics and so regression analyses were conducted on a reduced sample which may not be representative of the entire study population. While this study included several potential confounding variables it did not include concurrently measured information on comorbidities (e.g. cardiovascular disease [58–60]), neuropsychological conditions (e.g. depression/anxiety [60–62]) or other RA disease features (pain [62, 63], fatigue [64], medication [65, 66]) which may influence cognitive function. This information is collected within the longitudinal RESIST study that is ongoing. Results from RESIST will provide more clarity on the association between inflammation, comorbidities and cognition.

Despite limitations this study has strengths and important implications. This is the largest study to date to investigate cognitive function in a population of adults with RA, thus adding valuable information to the existing evidence base surrounding the prevalence of cognitive impairment in these patients. Cognitive impairment in RA can have significant impact on ability to plan and successfully complete daily activities and is also important for adhering to treatment regimens. This study suggests that routine cognitive screening in rheumatology clinics may be a useful tool in RA disease management. Additionally, higher levels of disease activity were associated with poorer cognitive performance, suggesting that the association between reduced cognitive function may be, at least in part, driven by the effects of systemic inflammation. This raises the intriguing possibility that controlling the systemic inflammatory response in RA using DMARDs might have benefits to cognitive function. This could also be transferable to other situations in which inflammation appears to drive cognitive decline such as AD.

## Conclusions

In this study, which is the largest to date that has investigated cognition in RA, we found that cognitive impairment was highly prevalent in RA and is partly driven by the effects of chronic systemic inflammation. This has implications for our understanding of how chronic inflammation may drive accelerated cognitive decline in the general population. RA disease activity is a potentially modifiable risk factor that may be targeted to slow or prevent cognitive decline.

## Supplementary Information

**Additional file 1.** Binary logistic regression predicting likelihood of cognitive impairment (scoring ≤27 on MoCA).

**Additional file 2.**

## Data Availability

Non-identifiable relevant raw data on which the conclusions of this paper rely is supplied as an Excel file in Supplementary material.

## Abbreviations

RA: Rheumatoid arthritis
ACR/EULAR: American College of Rheumatology/European League Against Rheumatism
UK: United Kingdom
MoCA: Montreal Cognitive Assessment
DAS28: Disease activity score
anti-CCP: Anti-cyclic citrullinated peptide
DMARDs: Disease Modifying Anti-Rheumatic Drugs
cs DMARDs: Conventional synthetic Disease Modifying Anti-Rheumatic Drugs
b DMARDs: Biologic Disease Modifying Anti-Rheumatic Drugs
ts DMARDs: Targeted synthetic Disease Modifying Anti-Rheumatic Drugs
AD: Alzheimer’s disease
RESIST: Rheumatoid arthritis medication and memory study
MCI: Mild cognitive impairment
TNFi: Tumour Necrosis Factor Inhibitor
STROBE: Strengthening the Reporting of Observational Studies in Epidemiology
BHSCT: Belfast Health and Social Care Trust
NHSCT: Northern Health and Social Care Trust
UHS: University Hospital Southampton NHS Foundation Trust
ESR: Erythrocyte sedimentation rate
CRP: C-reactive protein
RF: Rheumatoid factor
NSAID: Non-steroidal anti-inflammatory drug
SD: Standard deviation
