# Supplementary material for: Prevalence of cognitive impairment in patients with rheumatoid arthritis: a cross sectional study
Source: BMC Psychiatry. 2022 Dec 9;22:777. doi: 10.1186/s12888-022-04417-w (PMC9733399; doi:10.1186/s12888-022-04417-w)
Supplement: Supplementary file 1 — Additional file 1. Binary logistic regression predicting likelihood of cognitive impairment (scoring ≤27 on MoCA). [file 12888_2022_4417_MOESM1_ESM.docx]

**Additional file 1**. Binary logistic regression predicting likelihood of cognitive impairment (scoring ≤27 on MoCA)

| Predictor | B | SE | p | OR (95% CI) |
| --- | --- | --- | --- | --- |
| Age | 0.06 | 0.02 | **.009^*^** | 1.06 (1.02, 1.11) |
| Sex |  |  |  |  |
| *Male (reference)* | - | - | - | - |
| *Female* | -0.44 | 0.37 | .239 | 0.65 (0.31, 1.34) |
| Education |  |  |  |  |
| *≤12 years (reference)* | - | - | - | - |
| *>12 years* | -0.11 | 0.34 | .755 | 0.90 (0.46, 1.76) |
| Years Diagnosed | -0.01 | 0.02 | .471 | 0.99 (0.95, 1.02) |
| Disease Severity |  |  |  |  |
| *Remission (DAS28≤2.6) (reference)* | - | - | - | - |
| *Low (2.61<DAS28≤3.2)* | -0.40 | 0.49 | .416 | 0.67 (0.25, 1.76) |
| *Moderate (3.21<DAS28≤5.1)* | 0.98 | 0.41 | **.016^*^** | 2.66 (1.20, 5.91) |
| *High (DAS28>5.1)* | 1.21 | 0.55 | **.028^*^** | 3.34 (1.14, 9.81) |
| RF |  |  |  |  |
| *Negative (reference)* | - | - | - | - |
| *Positive* | -0.02 | 0.45 | .964 | 0.98 (0.41, 2.35) |
| Anti-CCP |  |  |  |  |
| *Negative (reference)* | - | - | - | - |
| *Positive* | 0.54 | 0.44 | .213 | 1.72 (0.73, 4.05) |
| RA Medication |  |  |  |  |
| *DMARD + TNFi (reference)* | - | - | - | - |
| *DMARD only* | 0.30 | 0.47 | .529 | 1.35 (0.53, 3.38) |
| *TNFi only* | 0.15 | 0.79 | .848 | 1.16 (0.25, 5.42) |
| Was ppt taking prednisolone |  |  |  |  |
| *No (reference)* | - | - | - | - |
| *Yes* | -0.42 | 0.73 | .564 | 0.66 (0.16, 2.74) |
| Was ppt taking NSAID |  |  |  |  |
| *No (reference)* | - | - | - | - |
| *Yes* | -1.28 | 0.46 | **.006^*^** | 0.28 (0.11, 0.69) |

Note. *Significant at the 5% level, *p*<0.05
